# PepFoundry: A Pipeline for Building Machine-Learning Ready Representations of Nonstandard Peptides Containing Cycles, Non-natural Residues, Polymer Units, and More

**DOI:** 10.1021/acs.jcim.5c02629

**Published:** 2026-01-13

**Authors:** Daniel Garzon Otero, Omid Akbari, Aneesh Mandapati, Camille Bilodeau

**Affiliations:** † 2358 University of Virginia, Chemical Engineering Department, 385 McCormick Road, Charlottesville, Virginia 22903, United States

## Abstract

Peptides featuring synthetic modifications, such as noncanonical amino acids, backbone modifications, cyclic structures, and polymer units have become central to modern drug design due to their enhanced stability and functional diversity. However, current machine learning (ML) approaches are restricted by challenges associated with transforming peptide sequences into atom-level representations, leading ML efforts to focus largely on datasets containing linear peptides comprised of standard residues. Here, we present PepFoundry, a Python package that handles peptide sequences beyond canonical amino acids and linear topologies by using SMILES strings in the CHUCKLES format. PepFoundry generates atom-mapped RDKit molecule objects, enabling the extraction of atom-level features, such as Morgan fingerprints and graph representations. We demonstrate its utility by processing a dataset of peptide sequences containing noncanonical amino acids and generating atomic level features for downstream property prediction. We show that atomic-level representations of peptides containing noncanonical amino acids consistently outperform sequence-level representations, regardless of model type. We additionally explore the representation of noncanonical peptides through latent space visualization and show that models with atomic-level information can effectively learn relationships between analogous sequences of l-peptides, d-peptides, and peptoids. This framework allows for the flexible incorporation of new amino acid chemistries, enabling existing ML methods to be straightforwardly applied to datasets of peptides containing nonstandard features. It also facilitates the rapid construction of customized peptide libraries and provides a scalable platform to accelerate ML-driven peptide discovery and optimization.

## Graphical abstract

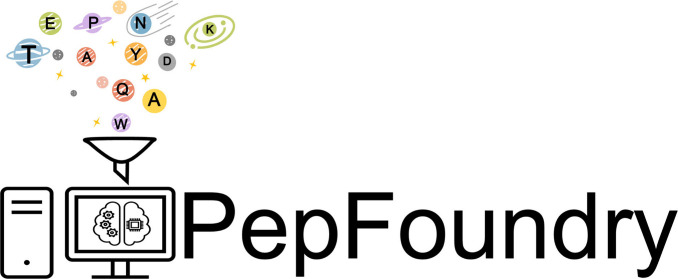

## 1 Introduction

Peptides with synthetic modifications, such as noncanonical amino acids (NCAAs), backbone modifications, cyclic structures, and polymer units, have become prevalent in peptide-based drug design, fueling a market worth over $40 billion^1^ and allowing access to molecules and materials with a range of useful properties and behaviors. For example, FDA-approved antimicrobial peptides (AMPs) often feature d-amino acid substitutions or cyclization, which enhance protease resistance and improve membrane permeability.^2–4^ Similarly, non-natural amino acid side chains can be used to fuse peptides with fluorescent labels, making it possible to design enzymes with superior catalytic properties.^5^ This growing building block variety opens up a vast design space, serving both to create major opportunities for innovation while also leading to significant challenges for systematic exploration of this design space.^6–8^ Machine learning (ML) has become a useful strategy to address these scientific challenges and is increasingly used to accelerate peptide discovery and optimization.^9^

Over the years, peptide modeling in ML has relied on sequence-based descriptors.^9^ Common approaches include using composition-based descriptors and physicochemical property encoding.^9,10^ These representations capture key features of natural residues and are simple to compute, enabling their use in many predictive tasks.^11–17^ For instance, Composition–Transition–Distribution^10^ (CTD) descriptors place amino acids into categories based on their physiochemical properties and then describe the overall peptide sequences based on the composition and distribution of these categories throughout the sequence. An alternative strategy is to represent peptides using Evolutionary Scale Model^18^ (ESM) embeddings, which extract embeddings from large language models trained on large, evolutionary protein databases. Importantly, both methods represent peptides as linear sequences of 20 natural residues. Therefore, they fall short when dealing with NCAAs, post-translational modifications, or nonlinear topologies, such as cyclic or stapled peptides. Moreover, they fail to represent chirality explicitly, resulting in poor generalizability across diverse, nonstandard peptide chemistries.^9,19^ As a result, current ML methods are incompatible with this growing class of molecules, and they are unable to predict their behavior or properties. Addressing this challenge requires strategies that can (1) generate ML-ready representations directly from the peptide sequences and (2) facilitate the flexible incorporation of new residues.

An alternative to these linear sequence-based strategies is to model each residue at the atomic-level. To illustrate this concept, consider 3-methyl phenylalanine, an NCAA that differs from the canonical amino acid (CAA) phenylalanine by a single methyl group (Figure 1). Conventional models treat these two amino acids as fully distinct residues, requiring either human-engineered descriptors or large amounts of data to learn their functional similarity. On the other hand, a more promising solution is to describe peptides at the atomic level, allowing the model to recognize structural similarities between phenylalanine and 3-methyl phenylalanine and to infer the properties of the latter without extensive training. In this way, models relying on one-hot encodings must learn relationships between amino acids purely from examples, whereas models with access to atomic-level information can innately recognize the degree of similarity among amino acids. Furthermore, this approach facilitates the modeling of nonlinear peptide systems by explicitly encoding each atom and its connectivity within the molecule.

**Figure 1:**
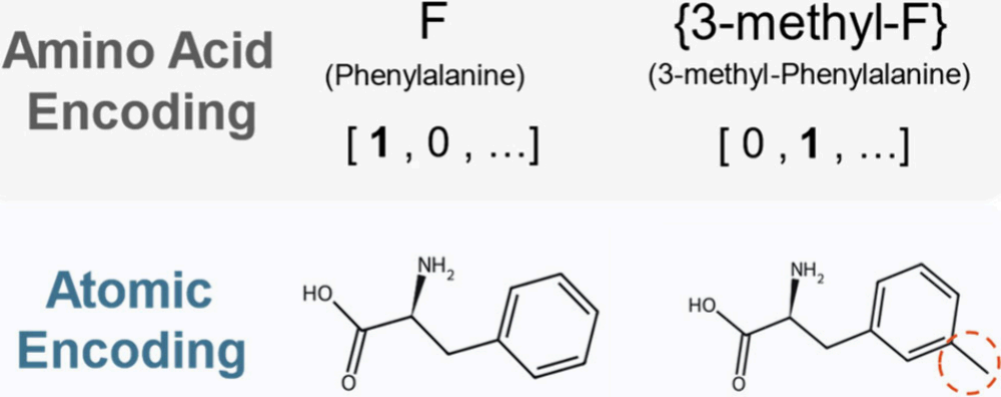
Comparison of amino-acid-level vs atomic-level representations. When incorporating noncanonical amino acids (NCAAs) using one-hot encoding methods, they are treated as entirely distinct, causing loss of information or restricting model learning.

To implement atom-level representations in practice, one must rely on robust computational tools capable of parsing and encoding peptide sequences. In this context, RDKit^20^ serves as the main Python package for molecular manipulation and is commonly used for small molecule ML. A given peptide sequence can be input to RDKit if it is converted into one of the traditional string-based representations as Simplified Molecular Input Line Entry System (SMILES)^21,22^ for atom-level chemical structures or Hierarchical Editing Language for Macromolecules (HELM)^23^ notation for sequences of amino acids. The package parses the string input and generates an RDKit molecule object, which plays a central role enabling the generation of ML-ready representations, as shown in Figure 2 (left).

**Figure 2:**
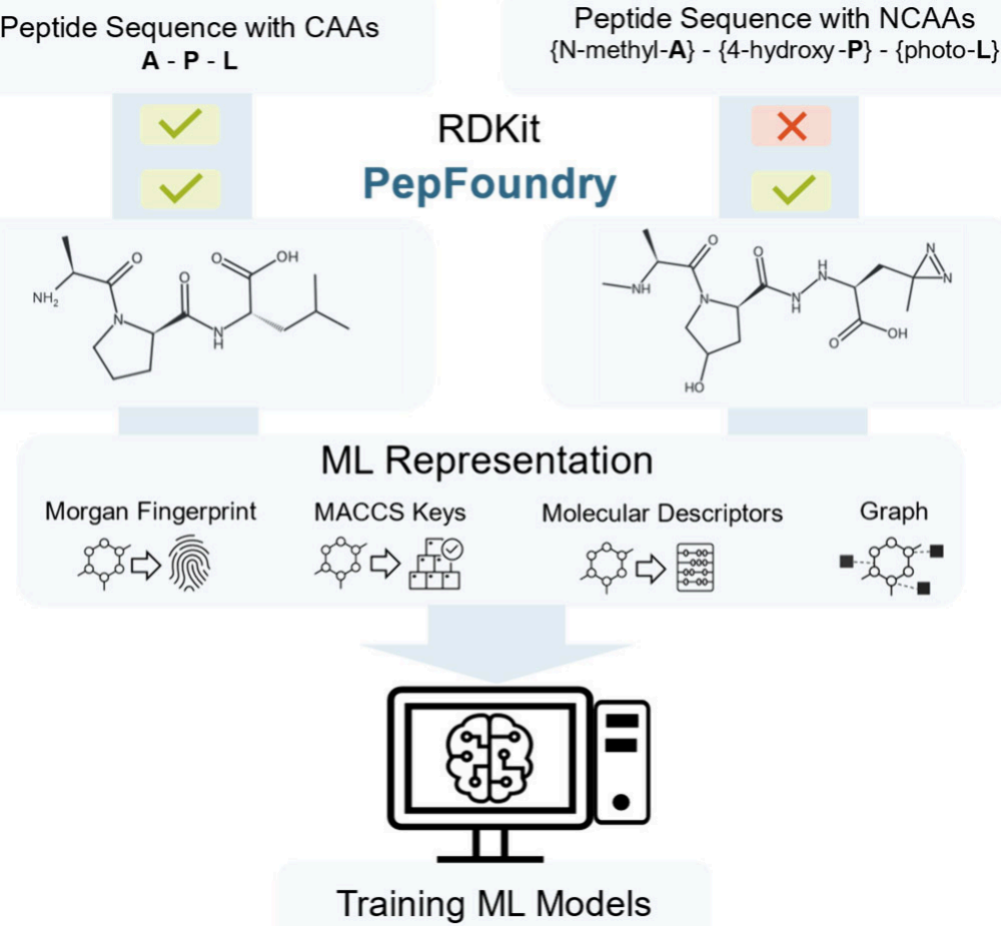
Limitations of RDKit in directly converting peptide sequences into RDKit molecule objects, especially when noncanonical amino acids (NCAAs) are present. PepFoundry overcomes these challenges, enabling the generation of atom-mapped RDKit molecule objects and unlocking diverse atomic-level representations for machine-learning applications.

However, in terms of parsing sequences at the amino acid level, strategies such as HELM are limited in RDKit. The current implementation is hard-coded and recognizes only a restricted set of monomers—specifically the CAAs, their d-isomers, and eight NCAAs.^20^ In parallel, frameworks to analyze peptides containing NCAAs have been proposed by constructing atomically detailed libraries of residues using the HELM notation.^24–30^ In these packages, each new residue must be explicitly defined in terms of its chemical properties, requiring the definition of structure data files (SDF).^31^ These files contain a list of residues, each of which requires a MOL file to be specified describing properties such as atomic composition, bonding structure, and associated metadata such as name, abbreviation, and SMILES representation. Overall, these methods are challenging to scale efficiently for new libraries because of the number of possible residues, the complexity of the syntax requirements, and the inclusion of nonlinear peptide architectures, limiting flexibility and ease of customization.^30,32^ Further, these packages are not designed to be used as an entry point to ML pipelines, instead providing tools such as similarity metrics and library generation.

To overcome these limitations, several frameworks have been developed to facilitate the peptide representation and conversion for ML applications. Some of these approaches support nonlinear peptide topologies, using strategies such as image-based encodings^33^ or simulation-derived features to capture structural complexity.^34–36^ While these methods broaden modeling capabilities for peptide representation and analysis, they are not intended for the direct parsing of peptide sequences for ML representation.^37^ Recently, this need has been addressed by p2smi,^37^ inspired by the method proposed by Duffy et al.,^38^ which enables FASTA files parsing and the direct conversion of peptide sequences into SMILES strings, allowing the creation of large-scale data sets supporting over 100 residues. However, p2smi relies on a fixed library of amino acids and NCAAs, and modifications to this library must be performed manually within the code.^37^ This limits flexibility for exploring custom residues or specific subsets of the peptide design space, highlighting the need for frameworks that support user-defined instance-specific libraries to efficiently navigate the exponentially growing chemical space of modified peptides.

Beyond library flexibility, SMILES encodes atom-level connectivity, and their length grows substantially for peptides compared to small molecules. In practice, this has led to scenarios where atom-level representations such as graphs can be less effective than sequence-based approaches,^39^ particularly when modeling NCAAs.^40^ To address this challenge, multiscale graph modeling strategies that combine atom-level and residue-level representations have proven effective, enabling models to capture both fine-grained chemical information and higher-order peptide structure simultaneously.^41–45^ Importantly, such multiscale methods require not only the atomic structure of the peptide encoded via SMILES, but also information about how to break the atomic structure into residues.

To address these limitations, we introduce PepFoundry, a Python package designed to convert peptide sequences into ML-ready representations for training models. Our package facilitates atom-level information extraction for nonstandard peptides and ensures adaptability for NCAAs, peptoid units, nonlinear peptides, and other nonstandard peptide features. PepFoundry leverages CHUCKLES-formatted SMILES to represent each residue at the atomic level,^46^ capturing features such as chirality and enabling seamless expansion to novel NCAAs. This format has been previously employed for de novo peptide design, where it was used to propose new sequences incorporating NCAAs.^47^ Extending this approach, PepFoundry allows each instance to be initialized with a user-defined residue library, allowing independent and flexible configurations tailored to specific applications. By manipulating SMILES strings, the package generates RDKit molecule objects and allows users to define custom tokens for residues in their data sets, supporting a wide array of peptide feature representations for ML applications (Figure 2).

In addition, PepFoundry is oriented to construct peptide graphs with atomic, bond features, and adjacency matrices, providing a useful framework for graph neural network applications. Beyond atomic graphs, PepFoundry supports hierarchical graph representations in which residue-level information can be explicitly mapped from the peptide RDKit molecule object using atom mapping, enabling multiscale learning strategies. The pipeline also allows users to encode stereochemical features, through dedicated atomic-level node attributes,^48^ and residue-level flags, ensuring that enantiomeric differences are preserved in the graph structure. All graph outputs facilitate integration with PyTorch Geometric, enabling direct application of GNN architectures. Moreover, the framework allows GPU usage, enabling faster graph construction.

Here, we demonstrate PepFoundry’s capabilities by processing a dataset of peptides containing NCAAs and generating atomic-level features for downstream property prediction. Using these features, we trained eight machine learning models, including hierarchical graph models that combine atom- and residue-level representations,^41^ highlighting how the package facilitates parsing of peptide sequences for model training. We also visualize the latent space of the embeddings and show that models with atomic-level information can effectively capture relationships among analogous sequences of l-peptides, d-peptides, and peptoids. This work enables the flexible incorporation of new amino acid chemistries and a scalable pipeline for ML-driven peptide discovery and optimization. Overall, PepFoundry allows researchers to systematically explore the peptide chemical space, streamline feature extraction, and facilitate ML-guided peptide design.

## 2 Methods

PepFoundry relies on the manipulation of SMILES strings, which describe molecules at the atomic level, to generate RDKit molecule objects. Importantly, the same molecule can be represented by multiple SMILES strings depending on which atom is chosen as the starting point and the path followed through the molecule. To simplify the mapping between SMILES and molecules, SMILES strings have been developed to have a canonical form that guarantees a unique textual signature for the molecule.^21,22^ However, it would be useful to be able to construct peptide SMILES through the concatenation of the SMILES strings of each individual amino acid, a functionality that is not present in the canonical representation of amino acids. To address this, PepFoundry represents peptide monomers using the CHUCKLES syntax, which deviates from the canonical SMILES syntax by defining the atoms of each amino acid in the following order: N-terminus, chiral carbon, and C-terminus.^46^ As an example, Figure 3 illustrates how the canonical SMILES of the amino acid l-Arginine can be rewritten in the CHUCKLES format while preserving the definition of the same molecule, even though the atom order in the string differs.

**Figure 3:**
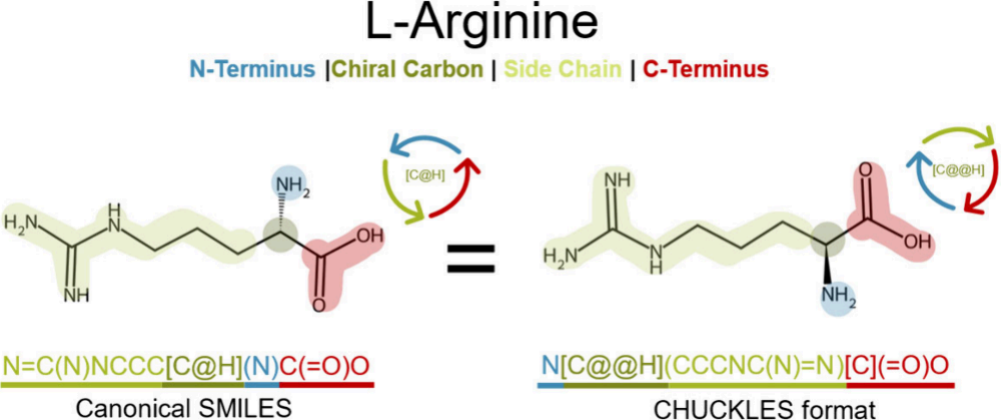
Canonical SMILES vs CHUCKLES representations of l-arginine. Using the CHUCKLES method, the molecule can be rewritten by reordering atoms while preserving its structure. Adjusting the chiral specification at the stereocenter is essential to maintain the correct molecular configuration.

Overall, PepFoundry enables ML of synthetically complex peptides by making atom-level representations of peptides readily available through combining SMILES manipulations with data manipulations available through RDKit. Specifically, (1) each amino acid is defined in CHUCKLES format, supporting both canonical and noncanonical residues; (2) peptide SMILES are generated by concatenating amino acid SMILES, (3) amino acids are identified and distinguished by atom-mapping of N- and C-terminal atoms to track peptide bonds, and (4) cycles are introduced through the inclusion of ring closure digits for cyclic sequences. PepFoundry then uses these atom-mapped peptide SMILES to generate RDKit molecule objects and further streamlines the creation of multiple ML-ready representations, such as molecule-level fingerprints, atom-level fingerprints, atom-level graphs, and amino acid-level graphs, enabling seamless integration into workflows for peptide property prediction, design, and other computational analyses. PepFoundry is available to the public along with detailed documentation and tutorials and can be installed directly via GitHub.

### 2.1 Implementing Peptide Representations with SMILES and CHUCKLES

Since SMILES strings describe molecular information at the atomic level, they enable representations of amino acids beyond the canonical forms. By adjusting this representation using the CHUCKLES method,^46^ (Figure 3), we can reduce peptide construction to simply concatenating the SMILES of each amino acid and removing the terminal atoms, specifically, the C-terminal oxygen and the N-terminal hydrogen, as illustrated in Figure 4a, which facilitates the translation of peptide sequences into peptide SMILES. This process requires as inputs (1) the peptide sequence, (2) the token defined for each amino acid in the peptide sequence (e.g., the one-letter amino acid code for canonical residues), and (3) the SMILES corresponding to each amino acid in the CHUCKLES format.

**Figure 4:**
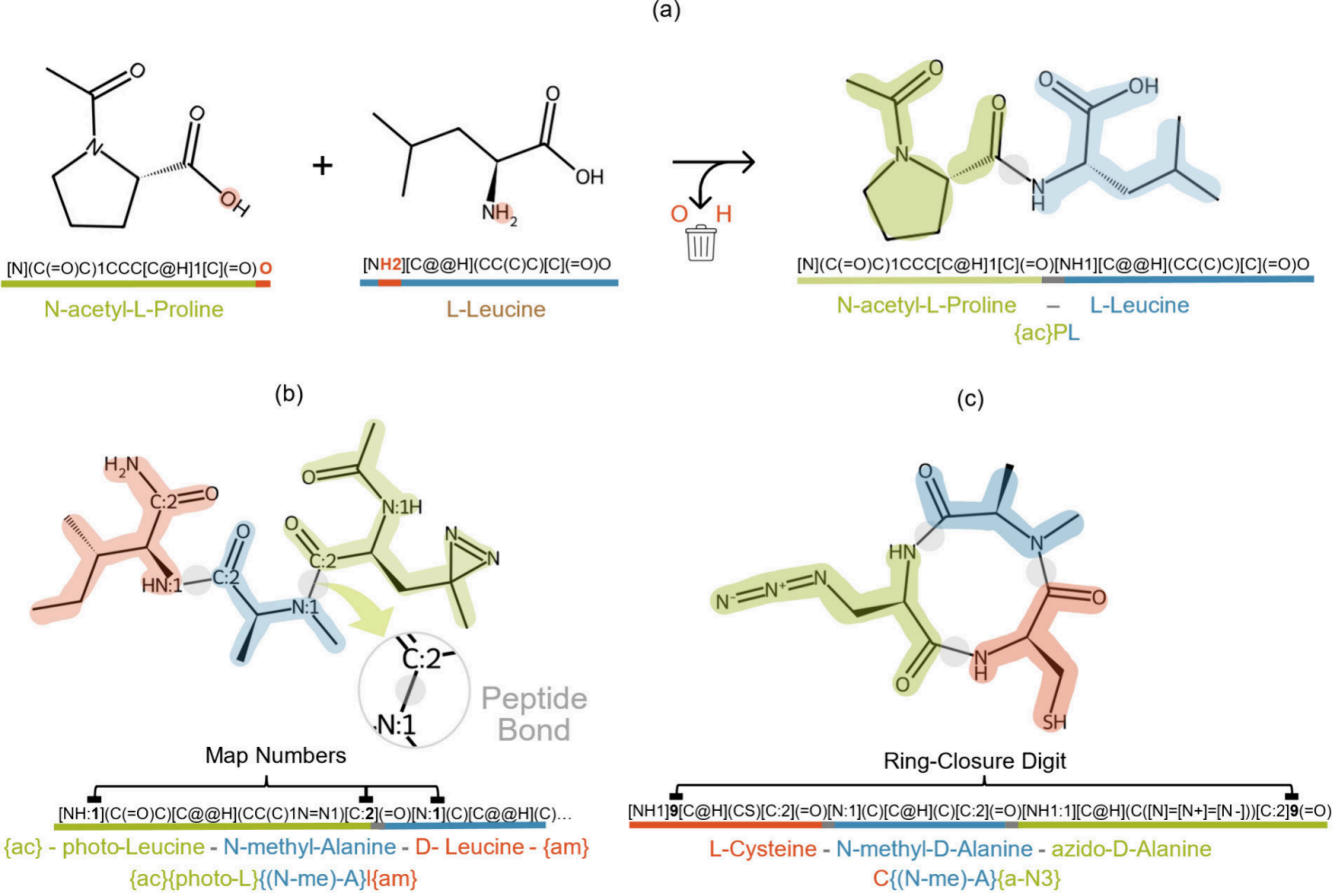
String-based manipulations in PepFoundry. (a) Construction of peptides using SMILES and CHUCKLES through concatenation of individual amino acid representations, ensuring correct atom valence in peptide bonds. (b) Peptide with mapped numbers on peptide bonds, illustrating how bond tracking can be defined from the amino acid string. (c) Conversion of a string-based representation into a cyclic peptide by introducing a ring-closure digit and ensuring proper valence between the atoms involved in the peptide bond linking the head and tail.

To illustrate this process, Figure 4a shows the example of constructing a peptide from N-acetyl-l-proline and l-leucine, where the SMILES of each amino acid was represented using the CHUCKLES method. The dipeptide can be translated into the resulting peptide SMILES by concatenating these two SMILES, removing the oxygen atom at the C-terminus of the first amino acid, and removing the hydrogen atom at the N-terminus of the second amino acid.^46^ Manipulating these strings through PepFoundry enables generation of the RDKit molecule object associated with the resulting dipeptide.

Importantly, this method of organizing atoms for amino acids can also be applied to peptoid units. In this implementation, each unit is described by its N-terminus, side chain, and C-terminus since these units do not possess a chiral α-carbon. In general, given the modularity of these systems, both peptides and peptoids can be treated as building blocks, allowing us to track the bonds that define each unit such as peptide bonds. To this end, in addition to arranging atoms using CHUCKLES, we implemented atom mapping numbers into the SMILES definition, as described in Figure 4b. We assign the number 1 to the nitrogen atom at the N-terminus and the number 2 to the carbon atom at the C-terminus. Consequently, it is possible to track the bonds between these two termini and thereby determine which atoms belong to each of the building blocks of each molecule. This is useful in situations where one wants to model information at the amino-acid level in addition to the atomic level as has been successfully done in multiple previous studies.^9,42,43^

Additionally, this process of concatenating and manipulating SMILES strings in the CHUCKLES format enables the construction of valid SMILES for cyclic peptides. For example, in head-to-tail cyclic peptides, a peptide bond is formed between the N-terminus of the first residue (head) and the C-terminus of the last residue (tail). To represent this linkage in SMILES, a ring-closure digit is added, as illustrated in Figure 4c. The cycle is created by first removing the oxygen atom from the C-terminus of the last amino acid and the hydrogen atom from the N-terminus of the first amino acid. This approach mirrors the amino acid concatenation process used for linear peptides but accounts for the formation of the cyclic structure.

Overall, PepFoundry provides a suite of methods for manipulating SMILES strings from sequence notation. To this end, we have built a database of 157 amino acid and peptoid units formatted following the CHUCKLES syntax. These manipulation methods can be applied to the provided amino acid library, or users can implement their own custom libraries, allowing them to add additional amino acids or customize the amino acid tokens (Figure 5). Furthermore, PepFoundry allows users to generate RDKit molecule objects and atomic graph representations, providing utilities for downstream ML applications. The documentation and examples associated with each utility is available in the package documentation on our GitHub

**Figure 5:**
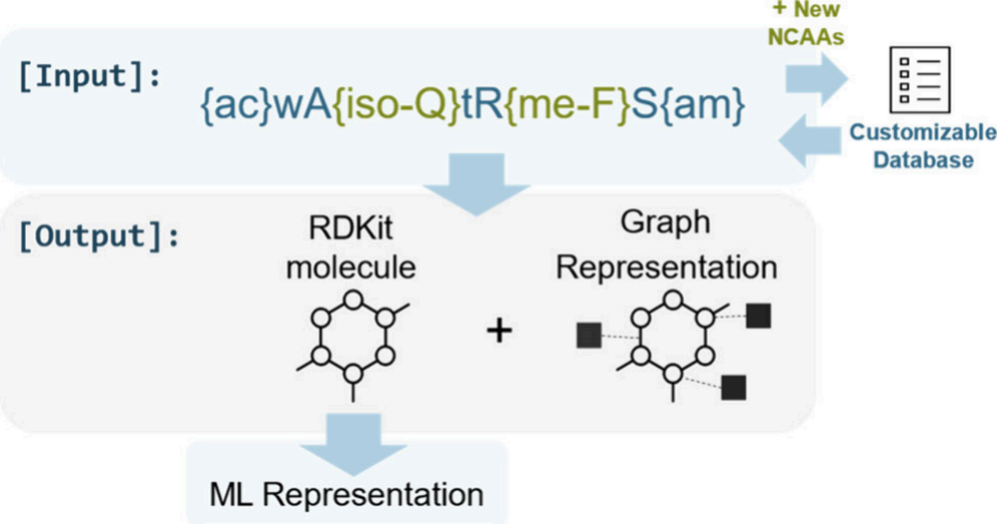
Illustration of the PepFoundry input and output. The package processes the input sequence and generates corresponding outputs, such as RDKit molecule objects or atomic graph representations.

### 2.2 Building a Customizable Amino Acid Database for Peptide Modeling

PepFoundry manipulates amino acid strings by relying on the tokens assigned to each residue and their associated SMILES strings in the CHUCKLES format. To streamline peptide modeling and prevent users from needing to convert monomer SMILES into CHUCKLES format, PepFoundry includes a comprehensive reference database of 157 entries, illustrated in Table 1. This database spans CAAs, d-amino acids, noncanonical side chains, peptoid units, end-capping chemistries (e.g., acetylation or amidation), and polymer units (e.g., PEG units). The chirality centers in the SMILES definitions have been verified to ensure consistency between the l- and d-type amino acids. Additionally, atom mapping numbers are assigned to the N- and C-terminal atoms, allowing tracking of peptide bonds formed when residues are concatenated. When available, CAS numbers are included and each residue is accompanied by a representative figure illustrating the residue structure, providing users with clear chemical reference and visualization (though these elements are not required for adding additional residues to the library).

**Table 1:**
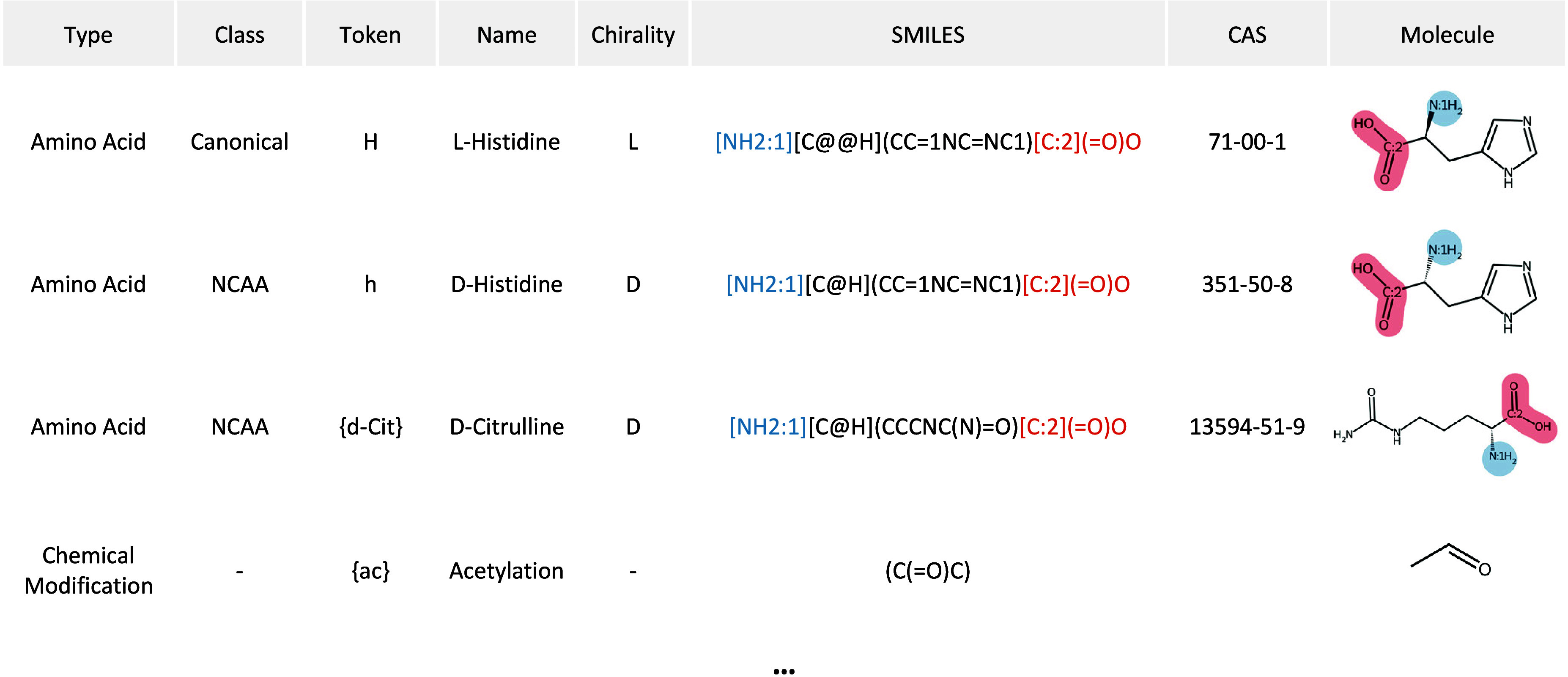
Database of Amino Acids and Modifications with Their Definitions and Representations^a^

a Each entry corresponds to either an amino acid or a modification. The token notation follows these conventions: uppercase letters for l-amino acids, lowercase letters for d-amino acids, and curly brackets (e.g., {Abc}) for NCAAs or chemical modifications. SMILES strings are defined using the CHUCKLES format, with atom-mapping numbers added for the C- and N-terminal atoms.

In this database, each residue or modification has an associated token: uppercase letters for l-type amino acids; lowercase letters for d-type amino acids; and the notation {Abc} for NCAAs and modifications, where Abc represents a symbol describing the residue within curly brackets. The database also encodes the chirality of each amino acid (l or d), which is used to define a flag that distinguishes between them in hierarchical graph processes where amino-acid-level information is required. For example, in the hierarchical graph model PepMNet,^9^ this flag is concatenated as [1,0] for l-type amino acids and [0,1] for d-type amino acids. This feature is deliberately designed to differentiate between l- and d-forms since their 2D graph representations would otherwise be equivalent.

PepFoundry is available with a default library that contains 157 residues with standard tokens. To ensure scalability for new residues and the corresponding flexibility, this library can be modified or replaced with a user-defined version. Users may provide an Excel file containing the desired amino acids as an optional argument when the PepFoundry class. Alternatively, users may modify the standard library to change the tokens to match the database syntax. However, this file must include, at a minimum, the same columns as the default library, such as the token and the CHUCKLES-formatted SMILES string for each amino acid, except for the CAS and Molecule image columns.

### 2.3 Graph Generation: From Amino Acid Sequences to Atomic Graphs

In recent years, graph-based peptide representation has been implemented with promising results for molecular property prediction.^9,43^ Describing atomic information and molecular connectivity facilitates the representation of peptides, including those containing NCAAs and nonlinear architectures. To streamline the implementation of this representation, PepFoundry generates the graph representation of the sequence from the peptide’s RDKit molecule object, including the node feature matrix, edge feature matrix, and adjacency matrix.

The dimensions of the node and edge features matrices for a given peptide are ***n*** x ***f***, where ***n*** is the number of atoms or bonds in the molecule, and ***f*** represents the atom-level features. The list of features used by PepFoundry is shown in Table S1. In general, these features are calculated based on the atoms present in the amino acids available in the default database; accordingly, the matrices have dimensions of (***n*** = atoms) × 24 for atom features and (***n*** = bonds) × 16 for bond features. If one intends to incorporate an NCAA whose atomic information is not described by the amino acids in the default database, then the package class must be instantiated with a custom database containing this new residue to capture and incorporate the corresponding atomic features.

Since PepFoundry also aims to maintain consistency with the stereochemistry of amino acids based on the SMILES strings in the database, users are given the option to include atom chirality configuration as an optional argument, thereby adding three additional columns to the node feature matrix: Sinister (S), Rectus (R), or Not Defined. Additionally, the adjacency matrix of size 2 × ***E***, where ***E*** is the number of edges or bonds in the molecule, can be obtained. Overall, these matrices enable the implementation of convolutional layers in packages for graph neural networks, such as PyTorch Geometric.^49^

Finally, for cases where atomic information must be combined within each amino acid (e.g., hierarchical methods^9,42,43^ ), a mapping vector is included that contains the information on which atom corresponds to each amino acid. With PepFoundry, we aim to facilitate the extraction of the parameters required for the application of graph-based neural networks to peptide properties, including NCAAs and cyclic architectures

## 3 Results and Discussion

We demonstrate the implementation of PepFoundry and its ability to facilitate the application of ML to peptide sequences containing NCAAs. We first trained eight ML models on a peptide property prediction task, where PepFoundry enabled the generation of the corresponding RDKit molecule objects for each sequence and, consequently, the extraction of atom-level molecular features such as Morgan fingerprints,^50^ MACCS keys,^51^ and molecular graph representations. We compared the performance of these atomic-level representations to sequence-level one-hot encodings. Second, we visualized the learned embeddings from one of the deep learning models to investigate the relationships between the peptide classes and their chemical structures within this latent space. We emphasize that these case studies serve to demonstrate that atomic-level representations are essential for peptides with complexities beyond canonical residues. Furthermore, they highlight PepFoundry’s ability to facilitate the generation of ML-ready representations.

### 3.1 Case Study 1: Antimicrobial Classification Using NCAA-Containing Sequences

To explore PepFoundry’s ability to facilitate the ML of nonstandard peptides, we selected antimicrobial peptide (AMP) classification as a model prediction task. AMPs are short cationic peptides with broad antimicrobial activity, making them attractive antibiotic alternatives^9^ and many FDA-approved examples incorporate d-amino acid substitutions or cyclization.^2,3^ We used the data sets employed by He et al., which include previously established peptide data sets^52–54^ as well as the NCAA data set newly constructed in their recent work.^43^ After removing duplicates, the training set comprised 61719 sequences, while the test set, obtained from the union of the test data sets reported in that study and deduplicated in the same way, contained 15114 sequences. Details on how the training set was further split into training and validation subsets are provided in Section S1.

We parsed the peptide sequences directly using PepFoundry, obtaining the corresponding RDKit molecule objects, from which Morgan fingerprints and MACCS keys were computed using standard RDKit functions. PepFoundry was also used to generate molecular graphs for each sequence, and all of these atomic-level representations were compared against a one-hot encoding of the 57 amino acids available in the data set from He et al. We note that we did not test other representations like CTD descriptors or ESM embeddings because these were not compatible with the noncanonical residues in our data set.

The resulting representations were then used to train seven ML models implemented with scikit-learn,^55^ as well as our previously published hierarchical graph model PepMNet,^9^ for which hyperparameters were kept fixed and not subject to further tuning. Hyperparameter tuning procedures for the remaining models are described in Section S1.

All atom-level representation methods, with the exception of MACCS keys, outperformed the sequence-level one-hot encoding across all evaluation metrics (Table 2). The relatively lower performance of MACCS keys is likely due to their limited ability to capture the full diversity and structural complexity of the amino acids in these data sets, as well as their low dimensionality (126 bits) compared to the 2048 bits of Morgan fingerprints. In contrast, Morgan fingerprints surpassed one-hot encoding, suggesting that richer atom-level information can enhance model performance. Importantly, these trends were consistent and independent of which type of machine learning method was used, illustrating that, for peptides containing NCAAs, atomic-level representations consistently outperform sequence-level representations.

**Table 2:** Performance of Different ML Models across Multiple Sequence Representations, Evaluated Using Three Metrics: Area under the Receiver Operating Characteristic Curve (AUC-ROC), Average Precision, and F1 Score^a^

Model	Featurization	F1	Avg Precision	ROC-AUC
SVM	One-Hot Encoding	0.699	0.772	0.878
	Morgan Fingerprints 2048	0.806	0.906	0.945
	MACCS Keys	0.606	0.652	0.792
RF	One-Hot Encoding	0.692	0.778	0.886
	Morgan Fingerprints 2048	0.811	0.901	0.940
	MACCS Keys	0.593	0.650	0.795
Decision Tree	One-Hot Encoding	0.630	0.510	0.780
	Morgan Fingerprints 2048	0.667	0.544	0.801
	MACCS Keys	0.571	0.544	0.761
Extra Trees	One-Hot Encoding	0.696	0.716	0.873
	Morgan Fingerprints 2048	0.817	0.905	0.942
	MACCS Keys	0.586	0.597	0.784
Gradient Boosting	One-Hot Encoding	0.692	0.789	0.886
	Morgan Fingerprints 2048	0.779	0.881	0.929
	MACCS Keys	0.603	0.675	0.802
kNN	One-Hot Encoding	0.668	0.703	0.865
	Morgan Fingerprints 2048	0.741	0.857	0.924
	MACCS Keys	0.582	0.578	0.773
MLP	One-Hot Encoding	0.649	0.731	0.843
	Morgan Fingerprints 2048	0.696	0.808	0.893
	MACCS Keys	0.581	0.643	0.776
PepMNet	Graph	**0.877**	**0.967**	**0.981**

a The models include Decision Tree, Extra Trees, Gradient Boosting, Multi-Layer Perceptron (MLP), Random Forest (RF), Support Vector Machine (SVM), k-Nearest Neighbors (kNN), and the hierarchical graph-based model PepMNet. The sequence representations evaluated are one-hot encoding, MACCS keys, and 2048-bit Morgan fingerprints and graph-based.

Among all of the trained ML models, the decision tree consistently showed the lowest performance, regardless of the featurization method used (Table 2). This is likely due to its limited capacity to model complex relationships between atomic- or residue-level features, especially given the diversity of residues and the dimensionality of the representations. As a result, the impact of the representation method is less pronounced in decision trees. For example, the performance differences between MACCS keys, Morgan fingerprints, and one-hot encoding are smaller compared to those observed in more complex models. In contrast, ensemble models such as random forests can better leverage detailed sequence and atom-level information and consequently show a larger performance gap between different representations. These results indicate that models with greater expressive power were more capable of exploiting the advantages of atom-level peptide information.

Finally, our previously developed hierarchical graph-based model, PepMNet, which learns peptide properties by aggregating atom-level information into amino acid-level features and then subsequently learning sequence-level representations, outperformed all other ML models and representation methods (Table 2). This illustrates that using an atomic graph representation and deriving residue-level features from it allow the model to effectively capture contributions at the amino acid level, regardless of whether they are CAAs or NCAAs. We note that full benchmarking of PepMNet has been described previously and is beyond the scope of this paper.^9^

### 3.2 Case Study 2: Visualizing Peptide Embeddings from the Hierarchical Graph Model

In the second case study, we were interested in exploring how models with access to atomic-level peptide information learned the similarities and differences between peptides containing structurally related residues. To visualize these relationships within the design space defined by the data set of He et al.,^43^ we extracted embeddings from the hierarchical graph model, PepMNet. This multiscale architecture performs graph convolutions first at the atomic level and subsequently at the amino acid level. The embeddings used in this study were obtained after amino-acid-level graph convolution, thereby incorporating information from both atomic and amino acid representations. Because PepMNet is an ensemble of five models, the final embedding for each sequence was constructed by concatenating the embeddings from all ensemble members.^9^ These embeddings were then projected with UMAP for dimensionality reduction,^56^ enabling visualization of the data set’s class distribution based on the learned representations. The results from the training data set are shown in Figure 6a,b.

**Figure 6:**
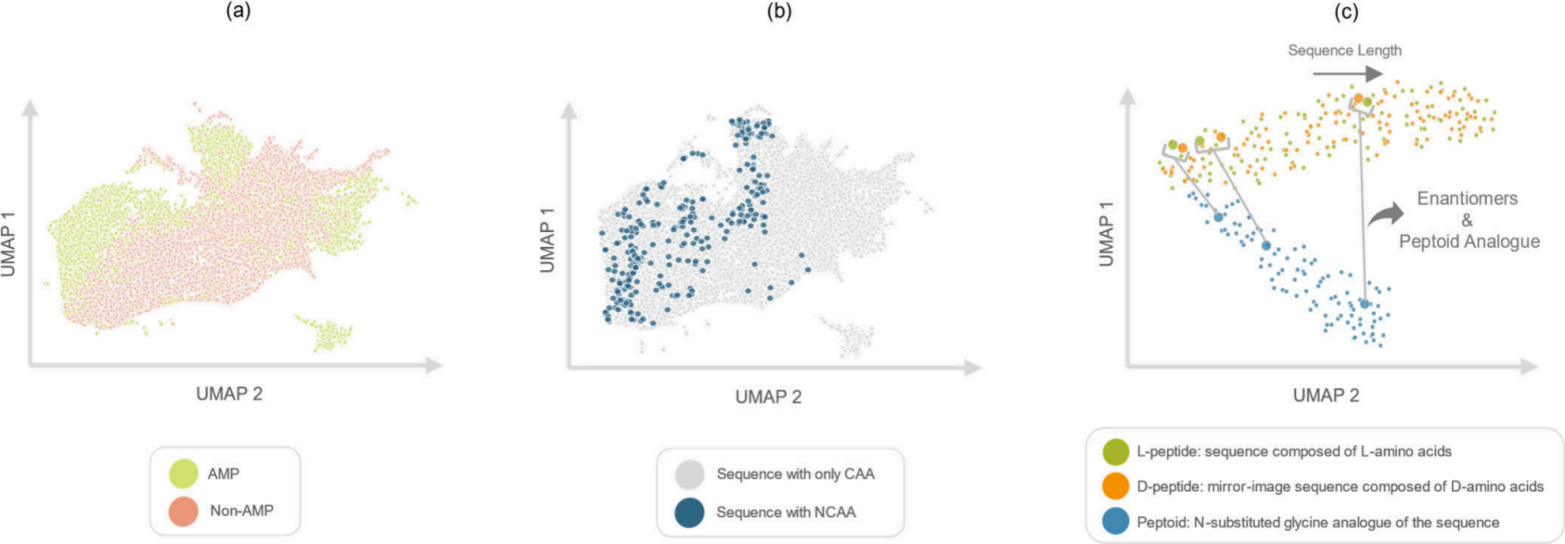
Visualization of peptide embeddings generated by the hierarchical graph-based model PepMNet by using UMAP. (a) Embedding space of antimicrobial peptide (AMP) sequences versus non-AMP sequences. (b) Embedding space of sequences containing canonical amino acids (CAAs) versus sequences containing noncanonical amino acids (NCAAs). (c) Embedding space of a random set of peptide sequences, showing each peptide alongside its corresponding d-peptide and peptoid analog.

As expected, Figure 6a shows that PepMNet effectively segregates the information captured between AMP (green) and non-AMP (red) classes. The regions containing each class remain distinct from each other, suggesting that the embeddings, which integrate atomic and amino acid information, learn meaningful features that distinguish AMPs from non-AMPs. For this process, we employed the mapping vectors generated by PepFoundry, which specify the correspondence between atoms and their respective amino acids in order to aggregate atomic information at the amino acid level.

A major advantage of representing peptides at the atomic level rather than at the amino acid level is that the model does not rely on predefined categorical representations of each residue but can instead learn atomic similarities and differences across residues. This atomic-level representation is particularly useful for handling sequences that include the NCAAs. To examine this, we analyzed how the graph model interprets sequences composed exclusively of canonical CAAs compared with those containing NCAAs. As shown in Figure 6b, The distribution of NCAA-containing sequences spans the learned representation space of both AMP and non-AMP classes, indicating that the model embeds canonical and noncanonical amino acids within a unified space by deriving their features from atomic structures rather than treating them as discrete categories.

Finally, we were interested in exploring how the model interpreted similarities and differences among l-peptides, d-peptides, and peptoids. To facilitate visualization of the model embedding space, we generated 100 random sequences with variable lengths between 5 and 30 l-residues. These sequences were then translated into versions in which all residues were d-amino acids, as well as corresponding versions composed of peptoid analog units. Proline and glycine were excluded from the sequences because they do not have peptoid analogs. We then projected the embeddings learned by PepMNet for each of these three classes of sequences to analyze how the model organizes them in the representation space (Figure 6c).

The hierarchical approach of PepMNet incorporates the amino acid flag obtained with PepFoundry (as described in Section 3.2) prior to the amino acid convolution stage, allowing the model to distinguish l ([1,0]) from d ([0,1]) residues. In the resulting embedding space, as shown in Figure 6c, we observed clustering of l and d sequences, as well as correspondence within each l–d analog pair. This correspondence is reflected in the Euclidean distances between embeddings of sequences in their l form and their corresponding d form, which ranged from 3.623 to 17.585, with an average distance of 9.556 ± 3.097 across all sequences, as reported in Figure S1­(a). Notably, this value is 13 times smaller than the distances obtained when comparing l sequences to their corresponding peptoid embeddings and d sequences to their corresponding peptoid embeddings, Figure S1b and c, respectively.

Building on this observation, the hierarchical model encodes the peptoid analogs separately from the l and d sequences while still reflecting their correspondence in the UMAP projection. Furthermore, as sequence length increases, the cluster containing the peptoid analogs becomes increasingly separated from the l and d clusters, resulting in a Spearman coefficient of 0.973 between Euclidean distance and sequence length, as shown in Figure S2. We hypothesize that this correlation between Euclidean distance and sequence length may be due to the accumulation of subtle atomic-level differences along the sequences, which can compound in the embeddings as sequences grow longer, amplifying the chemical and structural distinctions between peptoid and amino acid units, as reflected in UMAP projection Figure 6c. While it is unclear whether this distinction leads to better property prediction, since no peptoid units were present in the original data set, it is well-known that peptoids behave differently from peptides and it is expected that their differences would scale with length.^57^ We therefore expect this separation in latent space to be practically useful for ML applications.

## 4 Conclusions

Peptides with synthetic modifications, such as NCAAs and cyclic structures, are increasingly important in drug design. While they expand the peptide design space and create opportunities for innovation, they also pose challenges for systematic exploration using machine learning (ML). Here, we introduce PepFoundry, a Python package for processing peptide sequences beyond canonical amino acids and linear architectures. PepFoundry relies on string-based manipulation of SMILES strings in the CHUCKLES format, facilitating the construction of noncanonical peptide systems while enabling easy scalability and customization.

PepFoundry generates RDKit molecule objects, allowing ML models to accurately process sequences containing NCAAs, cyclic peptides, and peptoid analogs. It overcomes the limitations of traditional sequence-based descriptors, improving the scalability and flexibility for incorporating new residues across diverse peptide chemistries. Furthermore, the package enables the creation of graph-based representations of the peptides. In the first case study, we showed that atom-level representations are more effective than amino acid-level representations for predicting antimicrobial activity, a model peptide task. While our examples focused on classification tasks, PepFoundry can be readily applied to other predictive tasks, including regression tasks. In the second case study, we explored how learned embeddings from a hybrid atom-level amino acid model could learn the similarities and differences between peptides containing related residues. Overall, we expect that this Python package will streamline the atomic representation of nonstandard peptides, accelerate thing modeling of noncanonical peptide systems and support faster discovery and optimization in peptide-based drug development.

## Supplementary Material



## Data Availability

PepFoundry, including installation instructions and usage tutorials, is publicly available on our GitHub repository https://github.com/BilodeauGroup/PepFoundry. All relevant documentation for installation, configuration, and example workflows is provided in the repository.

## Notes

Atomic features and amino acid flags used by PepFoundry (Table S1); Optimal hyperparameters for each machine-learning model (Table S2); Performance of machine-learning models across multiple sequence representations (Table S3); Hyperparameter tuning procedures for machine-learning models; Analysis of Euclidean distances between L-, D- and peptoid embeddings; Relationship between peptide sequence length and Euclidean distances in latent space (Figures S1 and S2); Supplementary references (PDF)

## References

[1] Chang L., Mondal A., Singh B., Martínez-Noa Y., Perez A. (2024). Revolutionizing peptide-based Drug Discovery: Advances in the post-AlphaFold
Era. WIREs Comput. Mol. Sci..

[2] Zhang Q. (2025). Antimicrobial
Peptides: From Discovery to Developmental Applications. Appl. Environ. Microbiol..

[3] Sadeeq M., Li Y., Wang C., Hou F., Zuo J., Xiong P. (2025). Unlocking
the Power of Antimicrobial Peptides: Advances in Production, Optimization,
and Therapeutics. Front. Cell. Infect. Microbiol..

[4] Gray V. P., Amelung C. D., Duti I. J., Laudermilch E. G., Letteri R. A., Lampe K. J. (2022). Biomaterials via
Peptide Assembly:
Design, Characterization, and Application in Tissue Engineering. Acta Biomaterialia.

[5] Drienovská I., Roelfes G. (2020). Expanding the Enzyme Universe with
Genetically Encoded
Unnatural Amino Acids. Nat. Catal.

[6] Capecchi A., Zhang A., Reymond J.-L. (2020). Populating
Chemical Space with Peptides
Using a Genetic Algorithm. J. Chem. Inf. Model..

[7] Hoffmann T., Gastreich M. (2019). The next Level in Chemical Space Navigation: Going
Far beyond Enumerable Compound Libraries. Drug
Discovery Today.

[8] Awale M., Visini R., Probst D., Arús-Pous J., Reymond J.-L. (2017). Chemical Space: Big Data Challenge
for Molecular Diversity. Chimia.

[9] Garzon
Otero D., Akbari O., Bilodeau C. (2025). PepMNet: A Hybrid Deep
Learning Model for Predicting Peptide Properties Using Hierarchical
Graph Representations. Mol. Syst. Des. Eng..

[10] Dubchak I., Muchnik I., Holbrook S. R., Kim S. H. (1995). Prediction of Protein
Folding Class Using Global Description of Amino Acid Sequence. Proc. Natl. Acad. Sci. U.S.A..

[11] Bhadra P., Yan J., Li J., Fong S., Siu S. W. I. (2018). AmPEP: Sequence-Based
Prediction of Antimicrobial Peptides Using Distribution Patterns of
Amino Acid Properties and Random Forest. Sci.
Rep.

[12] Lawrence T. J., Carper D. L., Spangler M. K., Carrell A. A., Rush T. A., Minter S. J., Weston D. J., Labbé J. L. (2021). amPEPpy
1.0: A Portable and Accurate Antimicrobial Peptide Prediction Tool. Bioinformatics.

[13] Geng A., Luo Z., Li A., Zhang Z., Zou Q., Wei L., Cui F. (2025). ACP-CLB: An
Anticancer Peptide Prediction Model Based on Multichannel
Discriminative Processing and Integration of Large Pretrained Protein
Language Models. J. Chem. Inf. Model..

[14] Ansari M., White A. D. (2023). Serverless Prediction of Peptide Properties with Recurrent
Neural Networks. J. Chem. Inf. Model..

[15] Ma C., Ren Y., Yang J., Ren Z., Yang H., Liu S. (2018). Improved Peptide
Retention Time Prediction in Liquid Chromatography through Deep Learning. Anal. Chem..

[16] Meier F., Köhler N. D., Brunner A.-D., Wanka J.-M. H., Voytik E., Strauss M. T., Theis F. J., Mann M. (2021). Deep Learning the Collisional
Cross Sections of the Peptide Universe from a Million Experimental
Values. Nat. Commun..

[17] Yang J., Gao Z., Ren X., Sheng J., Xu P., Chang C., Fu Y. (2021). DeepDigest:
Prediction of Protein Proteolytic Digestion with Deep
Learning. Anal. Chem..

[18] Rives A., Meier J., Sercu T., Goyal S., Lin Z., Liu J., Guo D., Ott M., Zitnick C. L., Ma J., Fergus R. (2021). Biological Structure
and Function Emerge from Scaling
Unsupervised Learning to 250 Million Protein Sequences. Proc. Natl. Acad. Sci. U.S.A..

[19] Zhou X., Liu G., Cao S., Lv J. (2025). Deep Learning for Antimicrobial Peptides:
Computational Models and Databases. J. Chem.
Inf. Model..

[20] RDKit: Open-Source Cheminformatics. https://www.rdkit.org.

[21] Weininger D. (1988). SMILES, a
Chemical Language and Information System. 1. Introduction to Methodology
and Encoding Rules. J. Chem. Inf. Comput. Sci..

[22] Weininger D., Weininger A., Weininger J. L. (1989). SMILES. 2. Algorithm for Generation
of Unique SMILES Notation. J. Chem. Inf. Comput.
Sci..

[23] Zhang T., Li H., Xi H., Stanton R. V., Rotstein S. H. (2012). HELM: A Hierarchical
Notation Language for Complex Biomolecule Structure Representation. J. Chem. Inf. Model..

[24] Zeng W.-F., Zhou X.-X., Willems S., Ammar C., Wahle M., Bludau I., Voytik E., Strauss M. T., Mann M. (2022). AlphaPeptDeep:
A Modular Deep Learning Framework to Predict Peptide Properties for
Proteomics. Nat. Commun..

[25] Bouwmeester R., Gabriels R., Hulstaert N., Martens L., Degroeve S. (2021). DeepLC Can.
Predict Retention Times for Peptides That Carry As-yet Unseen Modifications. Nat. Methods.

[26] Capecchi A., Probst D., Reymond J.-L. (2020). One Molecular
Fingerprint to Rule
Them All: Drugs, Biomolecules, and the Metabolome. J. Cheminform.

[27] Orsi M., Reymond J.-L. (2024). One Chiral Fingerprint to Find Them
All. J. Cheminform.

[28] Chen R., You Y., Liu Y., Sun X., Ma T., Lao X., Zheng H. (2025). Deep-Learning-Based
Approaches for Rational Design of Stapled Peptides
With High Antimicrobial Activity and Stability. Microbial Biotechnology.

[29] Orsi M., Reymond J. (2025). Navigating a 1E+60
Chemical Space of Peptide/Peptoid
Oligomers. Molecular Informatics.

[30] Han H., Yeom M. S., Choi S. (2025). Hybridization
of SMILES and Chemical-Environment-Aware
Tokens to Improve Performance of Molecular Structure Generation. Sci. Rep.

[31] Ochoa R., Deibler K. (2025). PepFuNN: Novo Nordisk Open-Source
Toolkit to Enable
Peptide in Silico Analysis. Journal of Peptide
Science.

[32] Mizuno T., Kikuchi Y., Yasuhiro Y., Nemoto S., Hiroyuki K. (2025). Impact of
SMILES Notational Inconsistencies on Chemical Language Model Performance. arXiv:2505.07139 [q-bio.QM].

[33] Wang Z., Chen Y., Shang Y., Yang X., Pan W., Ye X., Sakurai T., Zeng X. (2025). MultiCycPermea: Accurate and Interpretable
Prediction of Cyclic Peptide Permeability Using a Multimodal Image-Sequence
Model. BMC Biol..

[34] Hui T., Secor M., Ho M. N., Bayaraa N., Lin Y.-S. (2025). Molecular
Dynamics (MD)-Derived Features for Canonical and Noncanonical Amino
Acids. J. Chem. Inf. Model..

[35] Baylon J. L., Ursu O., Muzdalo A., Wassermann A. M., Adams G. L., Spale M., Mejzlik P., Gromek A., Pisarenko V., Hancharyk D., Jenkins E., Bednar D., Chang C., Clarova K., Glick M., Bitton D. A. (2022). PepSeA:
Peptide Sequence Alignment and Visualization Tools to Enable Lead
Optimization. J. Chem. Inf. Model..

[36] Wang Z., Wu J., Zheng M., Geng C., Zhen B., Zhang W., Wu H., Xu Z., Xu G., Chen S., Li X. (2024). StaPep: An
Open-Source Toolkit for Structure Prediction, Feature Extraction,
and Rational Design of Hydrocarbon-Stapled Peptides. J. Chem. Inf. Model..

[37] Feller A. L., Wilke C. O. (2025). P2smi: A Python
Toolkit for Peptide FASTA-to-SMILES
Conversion and Molecular Property Analysis. arXiv:2505.00719 [q-bio.BM].

[38] Duffy F. J., Verniere M., Devocelle M., Bernard E., Shields D. C., Chubb A. J. (2011). CycloPs: Generating
Virtual Libraries of Cyclized and
Constrained Peptides Including Nonnatural Amino Acids. J. Chem. Inf. Model..

[39] Ruiz
Puentes P., Henao M. C., Cifuentes J., Muñoz-Camargo C., Reyes L. H., Cruz J. C., Arbeláez P. (2022). Rational Discovery
of Antimicrobial Peptides by Means of Artificial Intelligence. Membranes.

[40] Fernández-Díaz R., Ochoa R., Hoang T. L., Lopez V., Shields D. (2025). How to Generalize
Machine Learning Models to Both Canonical and Non-Canonical Peptides. ChemRxiv.

[41] Garzon
Otero D., Akbari O., Bilodeau C. (2025). PepMNet: A Hybrid Deep
Learning Model for Predicting Peptide Properties Using Hierarchical
Graph Representations. Mol. Syst. Des. Eng..

[42] Zhang R., Wu H., Liu C., Yang Q., Xiu Y., Li K., Chen N., Wang Y., Wang Y., Gao X., Zhou F. (2025). PepLand: A
Large-Scale Pre-Trained Peptide Representation Model for
a Comprehensive Landscape of Both Canonical and Non-Canonical Amino
Acids. Brief. Bioinform..

[43] He Y., Song X., Wan H., Zhao X. (2025). AmpHGT: Expanding Prediction
of Antimicrobial Activity in Peptides Containing Non-Canonical Amino
Acids Using Multi-View Constrained Heterogeneous Graph Transformer. BMC Biol..

[44] Jin, W. ; Barzilay, R. ; Jaakkola, T. Hierarchical Generation of Molecular Graphs Using Structural Motifs. Proceedings of the 37th International Conference on Machine Learning; PMLR, 2020.

[45] Ye W., Li J., Cai X. (2025). Mfgnn: Multi-Scale Feature-Attentive
Graph Neural Networks
for Molecular Property Prediction. J. Comput.
Chem..

[46] Siani M. A., Weininger D., Blaney J. M. (1994). CHUCKLES: A Method for Representing
and Searching Peptide and Peptoid Sequences on Both Monomer and Atomic
Levels. J. Chem. Inf. Comput. Sci..

[47] Geylan G., Janet J. P., Tibo A., He J., Patronov A., Kabeshov M., Czechtizky W., David F., Engkvist O., De Maria L. (2025). PepINVENT: Generative
Peptide Design beyond Natural
Amino Acids. Chem. Sci..

[48] Xu H., Lin J., Zhang D., Mo F. (2023). Retention Time Prediction for Chromatographic
Enantioseparation by Quantile Geometry-Enhanced Graph Neural Network. Nat. Commun..

[49] Fey M., Lenssen J. E. (2019). Fast Graph Representation Learning with PyTorch Geometric. arXiv1903.02428.

[50] Morgan H. L. (1965). The Generation
of a Unique Machine Description for Chemical Structures-A Technique
Developed at Chemical Abstracts Service. J.
Chem. Doc..

[51] Durant J. L., Leland B. A., Henry D. R., Nourse J. G. (2002). Reoptimization of
MDL Keys for Use in Drug Discovery. J. Chem.
Inf. Comput. Sci..

[52] Xu J., Li F., Leier A., Xiang D., Shen H.-H., Marquez
Lago T. T., Li J., Yu D.-J., Song J. (2021). Comprehensive
Assessment of Machine Learning-Based Methods for Predicting Antimicrobial
Peptides. Briefings in Bioinformatics.

[53] Pinacho-Castellanos S.
A., García-Jacas C. R., Gilson M. K., Brizuela C. A. (2021). Alignment-Free
Antimicrobial Peptide Predictors: Improving Performance by a Thorough
Analysis of the Largest Available Data Set. J. Chem. Inf. Model..

[54] Cordoves-Delgado G., García-Jacas C. R. (2024). Predicting
Antimicrobial Peptides
Using ESMFold-Predicted Structures and ESM-2-Based Amino Acid Features
with Graph Deep Learning. J. Chem. Inf. Model..

[55] Pedregosa F., Pedregosa F., Varoquaux G., Varoquaux G., Org N., Gramfort A., Gramfort A., Michel V., Michel V., Fr L., Thirion B., Thirion B., Grisel O., Grisel O., Blondel M., Prettenhofer P., Prettenhofer P., Weiss R., Dubourg V., Dubourg V., Vanderplas J., Passos A., Tp A., Cournapeau D. (2011). Scikit-Learn:
Machine Learning in Python. JMLR.

[56] McInnes L., Healy J., Melville J. (2020). UMAP: Uniform
Manifold Approximation
and Projection for Dimension Reduction. arXiv.1802.03426.

[57] Bonvin E., Personne H., Paschoud T., Reusser J., Gan B.-H., Luscher A., Köhler T., Van Delden C., Reymond J.-L. (2023). Antimicrobial Peptide-Peptoid Hybrids
with and without
Membrane Disruption. ACS Infect. Dis..

