# Supplementary material for: PepFoundry: A Pipeline for Building Machine-Learning Ready Representations of Nonstandard Peptides Containing Cycles, Non-natural Residues, Polymer Units, and More
Source: J Chem Inf Model. 2026 Jan 13;66(2):1264–73. doi: 10.1021/acs.jcim.5c02629 (PMC12848965; doi:10.1021/acs.jcim.5c02629)
Supplement: Supplementary file 1 [file ci5c02629_si_001.pdf]

Supporting Information

PepFoundry: A Pipeline for Building Machine-Learning Ready Representations of Non-Standard Peptides Containing Cycles, Non-natural Residues, Polymer Units and More

Authors: Daniel Garzon Otero[1], Omid Akbari[1], Aneesh Mandapati[1], Camille Bilodeau[1],[*†]
[1]University of Virginia, Chemical Engineering Department, 385 McCormick Road, Charlottesville, VA 22903
[*]Corresponding Author: cur5wz@virginia.edu
[†]Permanent address: 385 McCormick Road, Charlottesville, VA 22903

Description of Supporting Information: Additional tables, figures, and analyses complementing the main manuscript, including atomic features, hyperparameter tuning details, performance of different featurization embeddings analysis, and references.

- **S1:** Hyperparameter tuning details for machine learning models
    - **Table S1:** Atomic features and amino acid flags used by PepFoundry
    - **Table S2**. Best Hyperparameters for each ML model
    - **Table S3:** Performance of different ML models across multiple sequence representations
- **S2:** Euclidean distances between L-, D-, and peptoid embeddings
    - **Figure S1:** Relationship between peptide sequence length and Euclidean distances in the latent space
    - **Figure S2:** Relationship between peptide sequence length and Euclidean distances in the latent space
- **References**

S1. Hyperparameter tuning details for machine learning models

Table S1. Atomic Features and Amino acid flag used by PepFoundry.

Feature	Type Feature	Description	Size
Atom type	Atom	Type of atom by atomic number	7
Aromaticity	Atom	Whether this atom is part of an aromatic system	2
Number of bonds	Atom	Number of bonds the atom is involved in	4
Number of Hydrogen Bonds	Atom	Number of bonded hydrogen atoms	4
Hybridization	Atom	SP, SP2, SP3	3
Implicit Valence	Atom	Implicit valence of each atom	4
Atom Chirality	Atom	Atom chiral configuration: R, S, None	3
Bond Type	Bond	Single, double, triple, or aromatic	4
In ring	Bond	Whether the bond is part of a ring	2

Conjugated	Bond	Whether the bond is conjugated	2
Aromaticity Bond	Bond	Whether the bond is aromatic	2
Valence Contribution i	Bond	Contribution of the bond to the valence of Atom *i*	3
Valence Contribution f	Bond	Contribution of the bond to the valence of Atom *f*	3
Amino Acid Chirality	Amino acid	Amino acid chirality label: L or D	2

Hyperparameter tuning for each machine learning model (ML) was performed using a randomized search strategy implemented with scikit-learn.[1] For models with tunable parameters, including Support Vector Machine (SVM), Random Forest (RF), Decision Tree, Extra Trees, Gradient Boosting, k-Nearest Neighbors (kNN), and Multi-Layer Perceptron (MLP). Reflecting commonly adjusted hyperparameters such as regularization strength, kernel type, number of estimators, maximum depth, learning rate, and network architecture. For each feature set (Morgan fingerprints, MACCS keys, and one-hot encoding), the training data were split into 80% training and 20% validation subsets, and RandomizedSearchCV was applied with three-fold cross-validation and 10 random hyperparameter combinations. For the MLP classifier, early stopping was enabled with a validation fraction of 0.2 to prevent overfitting. The best-performing hyperparameter combination for each model and feature set was selected based on the F1 score on the validation data. All models were then retrained on the training data and evaluated on a held-out test set using Accuracy, F1 score, average precision, and ROC-AUC. The optimized hyperparameters are reported in Table S2.

Table S2. Best Hyperparameters for each ML model

Model	Best Hyperparameters
RF	n_estimators=100, min_samples_split=2
Decision Tree	max_depth=20, min_samples_split=2, criterion='entropy'
Extra Trees	n_estimators=100, min_samples_split=2
Gradient Boosting	n_estimators=300, learning_rate=0.1, max_depth=7
kNN	n_neighbors=10, weights='distance', metric='manhattan'
MLPC	hidden_layer_sizes=(128,64), activation='relu', alpha=0.001, learning_rate_init=0.001, max_iter=500
SVM	kernel='rbf', C=10, gamma=0.1, probability=True

For PepMNet employed the hierarchical graph neural network architecture as described in the paper,[2] consisting of two atom-level NNConv layers[3,4] (hidden dimensions 20 each), followed by an amino acid-level ARMAConv[5] layer (hidden dimension 50). The network concludes with three fully connected layers (hidden dimensions 100, 20, and 10) and a final output layer, with ReLU activations.

Table S3. Performance of different ML models across multiple sequence representations, evaluated using three metrics: area under the receiver operating characteristic curve (AUC-ROC), average precision, and F1 score. The models include Decision Tree, Extra Trees, Gradient Boosting, Multi-Layer Perceptron (MLP), Random Forest (RF), Support Vector Machine (SVM), k-Nearest Neighbors (kNN), and the hierarchical graph-based model PepMNet. The sequence representations evaluated are one-hot encoding, MACCS keys, 1024 and 2048-bit Morgan fingerprints, and graph-based.

Model	Featurization	F1	Avg. Precision	ROC-AUC
SVM	One-Hot Encoding	0.699	0.772	0.878
	Morgan Fingerprints 1024	0.799	0.901	0.943
	Morgan Fingerprints 2048	0.806	0.906	0.945
	MACCS Keys	0.606	0.652	0.792
RF	One-Hot Encoding	0.692	0.778	0.886
	Morgan Fingerprints 1024	0.801	0.895	0.937
	Morgan Fingerprints 2048	0.811	0.901	0.940
	MACCS Keys	0.593	0.650	0.795
Decision Tree	One-Hot Encoding	0.630	0.510	0.780
	Morgan Fingerprints 1024	0.654	0.529	0.793
	Morgan Fingerprints 2048	0.667	0.544	0.801
	MACCS Keys	0.571	0.544	0.761
Extra Trees	One-Hot Encoding	0.696	0.716	0.873
	Morgan Fingerprints 1024	0.809	0.904	0.942
	Morgan Fingerprints 2048	0.817	0.905	0.942
	MACCS Keys	0.586	0.597	0.784
Gradient Boosting	One-Hot Encoding	0.692	0.789	0.886
	Morgan Fingerprints 1024	0.772	0.875	0.927
	Morgan Fingerprints 2048	0.779	0.881	0.929
	MACCS Keys	0.603	0.675	0.802
kNN	One-Hot Encoding	0.668	0.703	0.865
	Morgan Fingerprints 1024	0.733	0.853	0.921
	Morgan Fingerprints 2048	0.741	0.857	0.924
	MACCS Keys	0.582	0.578	0.773
MLP	One-Hot Encoding	0.649	0.731	0.843
	Morgan Fingerprints 1024	0.669	0.793	0.881
	Morgan Fingerprints 2048	0.696	0.808	0.893
	MACCS Keys	0.581	0.643	0.776
PepMNet	Graph	**0.877**	**0.967**	**0.981**

S2. Euclidean distances between L, D, and peptoid embeddings

(a) (b) (c)

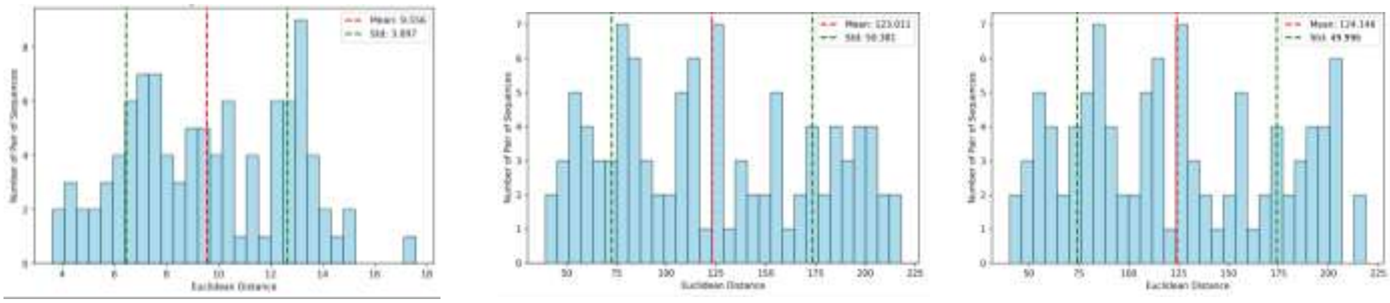

Figure S1. Histograms of Euclidean distances between latent embeddings of sequences. (a) L and D sequences, (b) L and corresponding peptoid sequences, (c) D and corresponding peptoid sequences. Red dashed lines indicate the mean, and green dashed lines indicate ±1 standard deviation.

(a) (b) (c)

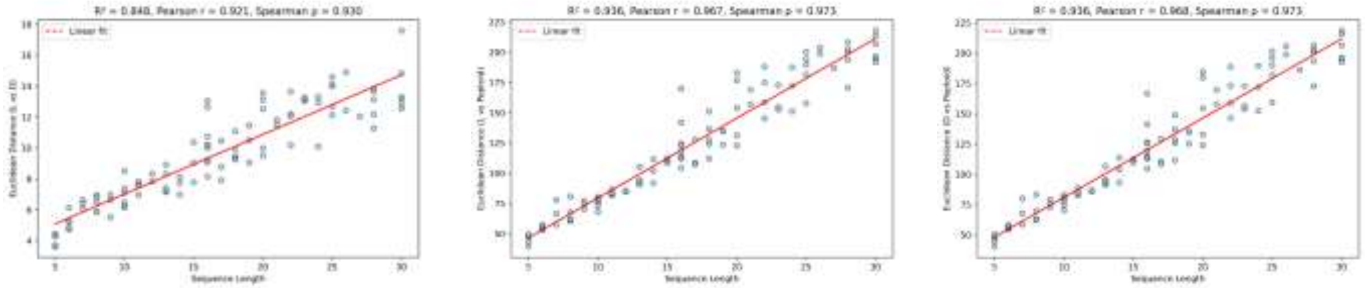

Figure S2. Relationship between peptide sequence length and Euclidean distances in the latent space. (a) Distance between L and D sequences, (b) Distance between L and corresponding peptoid sequences, (c) Distance between D and corresponding peptoid sequences. Correlation metrics including R^2, Pearson r, and Spearman ρ are reported in the figure title.

References

(1) Pedregosa, F.; Varoquaux, G.; Gramfort, A.; Michel, V.; Thirion, B.; Grisel, O.; Blondel, M.; Müller, A.; Nothman, J.; Louppe, G.; Prettenhofer, P.; Weiss, R.; Dubourg, V.; Vanderplas, J.; Passos, A.; Cournapeau, D.; Brucher, M.; Perrot, M.; Duchesnay, É. Scikit-Learn: Machine Learning in Python. arXiv June 5, 2018. http://arxiv.org/abs/1201.0490 (accessed 2023-07-16).

(2) Garzon Otero, D.; Akbari, O.; Bilodeau, C. PepMNet: A Hybrid Deep Learning Model for Predicting Peptide Properties Using Hierarchical Graph Representations. *Mol. Syst. Des. Eng.* **2025**, 10.1039.D4ME00172A. https://doi.org/10.1039/D4ME00172A.

(3) Simonovsky, M.; Komodakis, N. Dynamic Edge-Conditioned Filters in Convolutional Neural Networks on Graphs. arXiv August 8, 2017. http://arxiv.org/abs/1704.02901 (accessed 2024-08-12).

(4) Gilmer, J.; Schoenholz, S. S.; Riley, P. F.; Vinyals, O.; Dahl, G. E. Neural Message Passing for Quantum Chemistry. arXiv June 12, 2017. http://arxiv.org/abs/1704.01212 (accessed 2024-08-12).

(5) Bianchi, F. M.; Grattarola, D.; Livi, L.; Alippi, C. Graph Neural Networks with Convolutional ARMA Filters. *IEEE Trans. Pattern Anal. Mach. Intell.* **2021**, 1–1. https://doi.org/10.1109/TPAMI.2021.3054830.
